# Subgroups of emotion dysregulation in adults: a latent profile analysis in a clinically heterogeneous population

**DOI:** 10.1186/s40479-025-00319-x

**Published:** 2025-12-04

**Authors:** Martin Blay, Amaury Durpoix, Mario Speranza, Roland Hasler, Rosetta Nicastro, Eleonore Pham, Eva Rüfenacht, Luisa Weiner, Sébastien Weibel, Martin Debbane, Nader Perroud

**Affiliations:** 1 ADDIPSY, Addictology and Psychiatry Oupatient Center, Santé Basque Développement group, Lyon, France; 2 Université Paris-Saclay, UVSQ, INSERM, Centre de recherche en Epidémiologie et Santé des Populations Team DevPsy, Villejuif, 94807 France; 3 University Hospitals of Strasbourg, Strasbourg, France; 4 Service of Psychiatric Specialties, Department of Psychiatry, University Hospitals of Geneva, Geneva, Switzerland; 5 University of Geneva, Geneva, Switzerland; 6 Laboratoire de Psychologie des Cognitions, Faculté de Psychologie, University of Strasbourg, 12 Rue Goethe, Strasbourg, 67000 France; 7 INSERM UMR-S 1329 STEP Strasbourg Translational Neuroscience & Psychiatry, Strasbourg, France; 8 Developmental Clinical Psychology Research Unit, Faculty of Psychology and Educational Sciences, University of Geneva, Geneva, Switzerland; 9 Research Department of Clinical, Educational and Health Psychology, University College London, London, UK

**Keywords:** Emotion dysregulation, Dimensional, Latent profile analysis, Psychotherapy, Transdiagnostic

## Abstract

**Background:**

Even if it was first described in borderline personality disorder, emotion dysregulation (ED) is increasingly recognized as a transdiagnostic and dimensional construct, best understood along a continuum of severity rather than as a discrete category. Prior cluster-analytic studies have consistently explored heterogeneity within this continuum by identifying patient subgroups, but most were conducted in populations defined by specific diagnostic categories, limiting their generalizability. To better capture ED’s core components, studies conducted in clinically heterogeneous adult populations are needed.

**Methods:**

In this context, we conducted a latent profile analysis on 349 adults referred for ED-related difficulties at a specialized outpatient clinic. Profiles were derived from the six subscales of the Difficulties in Emotion Regulation Scale. Patients were assessed on sociodemographic variables, emotion regulation difficulties and strategies, mentalizing abilities, comorbid psychopathology, and well-being.

**Results:**

A two-class solution was retained. Subgroups did not align with any specific diagnostic category but rather emerged along a severity continuum. Cluster 1 (*n* = 267) was characterized by more severe ED in every domain, higher use of non-adaptive regulation strategies, lower self-mentalizing abilities, greater comorbid psychopathology, and lower well-being. Cluster 2 (*n* = 82) displayed comparatively preserved emotion regulation abilities and strategies, higher self-mentalizing, fewer comorbidities, and greater well-being.

**Conclusions:**

Our findings provide further evidence for the existence and characterization of subgroups in patients with ED, reflecting gradations along a severity continuum. While exploratory, they also suggest that core mechanisms targeted by DBT and MBT (i.e., maladaptive emotion regulation strategies and self-mentalizing abilities) may be relevant therapeutic targets across a broader transdiagnostic spectrum.

**Supplementary Information:**

The online version contains supplementary material available at 10.1186/s40479-025-00319-x.

## Background

Emotion dysregulation (ED) can be defined as persistent difficulties in emotion regulation, including identification and acceptance of emotions, abilities to modulate emotional arousal or to regulate emotion-related behavioral reactions, leading to impairment in developmental goals in short and/or long-term time frame [1–4]. The first popular conceptualization on how ED develops and expresses was proposed by Marsha Linehan in borderline personality disorder (BPD) [5]. Linehan described ED as the result of the interaction of a genetic emotional vulnerability (that includes lower threshold for emotion triggering, a larger intensity of emotions, and a slower decrease after emotional activation) with an invalidating environment (defined as an environment persistently ignoring, disregarding or punishing emotion expression). Since Linehan’s pioneer works, numerous other models have been developed, arising from either psychological research on emotions [2, 4] or from clinical observations in patients with BPD. An example arising from the latter is the mentalizing model, that describes emotion dysregulation as a consequence of impaired mentalizing abilities (i.e., the ability to understand and make sense of emotions, beliefs, intentions, thoughts, and motivations that drive our own behaviors as well as those of other) [6]. These models (notably Linehan’s or Bateman & Fonagy’s conceptualization of ED) have played crucial role in shaping clinical approaches for patients with BPD (dialectical behavioral therapy – DBT - and mentalization-based therapy - MBT, respectively), and have allowed tremendous advances in the care of this former difficult-to-treat population [5, 7, 8].

Even if ED was first described in BPD (which explains why it is mainly confined to the BPD diagnosis in current categorical classifications), it is now considered a transdiagnostic construct that can be found in various psychiatric disorders [1, 9, 10]. This issue, among others, may explain the high heterogeneity of patients suffering from ED, with some presenting clear borderline psychopathology, some displaying different dimensions (such as hyperactivity, post-traumatic stress, pathological narcissism, or autism spectrum disorder), and some exhibiting multiple dimensions simultaneously [1]. When overlooked, this high heterogeneity can impair the diagnostic and treatment processes, notably by restricting ED to BPD which may lead to missing other important symptomatic dimensions [1]. This observation has prompted clinicians and researchers to consider emotion dysregulation from a dimensional perspective, like many other mental health issues [11, 12], with direct consequences in terms of clinical practice and evidence-based treatments. For example, recent adaptations of DBT and MBT have been developed for other disorders associated with ED (e.g., autism spectrum disorder, attention deficit hyperactive disorder…) [13–16]. These efforts rely on the rationale that emotion dysregulation, as a shared dimension across conditions, should be the primary focus of intervention rather than the diagnostic category itself [1]. Such approach aligns with the growing interest on transdiagnostic treatments targeting core processes underlying multiple disorders, in order to improve both specificity and generalizability of therapeutic strategies [17]. However, to further advance on transdiagnostic ED treatment, we need a more precise understanding of the ED dimension itself. Notably, it seems essential to examine whether distinct subgroups of patients can be identified within ED and, if so, to describe their specific psychopathological profiles. This would allow for a more accurate characterization of its components and would support the development of more tailored interventions that go beyond “one-size-fits-all” treatment programs [18].

For this identification, several statistical methods, such as cluster analysis, can be helpful [19]. Using the following search equation: (“cluster*” OR “latent profile*”) AND (“DERS” OR “difficulties in emotion regulation scale”), we conducted literature research on PubMed and WebOfScience databases to identify such studies already published in ED research. We included every study (clinical or non-clinical) with cross-sectional cluster or latent profile analyses conducted on Difficulties in Emotion Regulation Scale (DERS) [3]. To note, the DERS assesses six dimensions of emotion regulation (lack of emotional awareness and clarity, goal-directed behavior, impulse control, non-acceptance of emotions, and limited access to regulation strategies), and is one of the most comprehensive and commonly used scale in emotion dysregulation research, including in cluster analytic studies [20]. However, the construction of emotion dysregulation is broad and can be assessed through numerous other conceptualizations and measures [21]. In the present review, we preferred to focus on a single, widely used tool allowed us to maintain clarity and comparability across studies, because including all the available tools would have made our review overly unspecific, and because it is also the instrument we used in the following study (which ensures consistency between our empirical work and the present review). We are aware that such selection yields some limitations that we will discuss later.

Overall, we found 73 results, with seven retained based on title and abstract, and six retained on full text [18, 22–26]. The six studies consistently identified subgroups arranged along a severity gradient, with a total of three clusters in five out of six studies [18, 22, 23, 25, 26]. In the two studies conducted in community samples, profiles typically include a low-ED group, an intermediate group characterized by selective vulnerabilities (e.g., poor emotional awareness [22]) or specific abilities (e.g., lower difficulties regulating positive emotions [26]), and a highly dysregulated group, with a concomitant gradient in terms of associated psychiatric difficulties. In clinical samples—such as patients with BPD [18, 25], binge-spectrum eating disorders [24], or adolescents with psychiatric disorders [23] —similar gradients emerge, most often with intermediate subgroups partially buffered by preserved capacities such as emotional acceptance [18] or awareness [25]. Once again, the more emotionally dysregulated the groups were, the more psychiatric comorbidities and psycho-social impairment were found. It is also interesting to note that, in the only transdiagnostic study, the subgroups found did not align with discrete self-reported diagnostic categories, underlying the limitations of diagnostic categories and the added value of considering ED dimensionally [23]. Altogether, these results suggest that subgroups of patients identified within the ED dimension follows a severity gradient associated with varying levels of comorbidity and functional impairment, and that some dimensions (notably emotional awareness [18, 22, 25]) seem to play an important role in this severity.

However, most of these studies were conducted in specific clinical populations (e.g., BPD, eating disorders), and the only transdiagnostic study was conducted in adolescent patients. This clearly limits the generalizability of the results to the global adult population of patients struggling with ED. Thus, to understand more about this dimension, we need studies conducted in clinically heterogeneous samples of adult patients suffering from ED. The present study aimed to fill this gap. We chose the exploratory stance given that no previous research of this kind has been conducted in this type of population. We had two main questions:

- Can sub-groups of patients be identified in a clinically heterogeneous population of adult patients consulting for ED, based on their emotion regulation difficulties?
- If yes, how many, and what are their characteristics and specificities?

## Methods

### Setting

The present study was conducted in a specialized outpatient unit for ED. Briefly, participants are referred by healthcare professionals or self-referred for the diagnosis and treatment of BPD, ADHD, or any other ED-related difficulties (such as emotional outbursts, repeated use of maladaptive emotion regulation strategies – including impulsive behaviors, self-harm, and suicidal attempts -, or interpersonal issues linked to emotion regulation difficulties). Inclusion criteria for this study were: 1°) Being at least 18 years old, 2°) Being referred to the unit, regardless of the diagnosis, and 3°) Providing written informed consent. Patients were included regardless of the reason for the consultation (diagnostic assessment only, diagnostic and treatment) or the mode of referral (self-referred, clinician-referred). Ethical approval was granted by the Geneva University Hospitals’ Ethics Committee (Protocol No. 2023 − 01391).

### Assessment

On their arrival in our unit, patients undergo several clinical evaluations. For categorical diagnoses, patients presenting with BPD symptoms at the initial evaluation are assessed with the Structured Clinical Interview for DSM-IV Personality Disorders adapted for DSM-5 [27], and patients presenting with ADHD symptoms are assessed using the ADHD Child Evaluation for Adults (ACE+) [28]. Thus, patients that do not undertake these structured evaluations are rated negative for the diagnosis. To note, these two diagnoses were the only categorically assessed, due to the specialty of our unit. Thus, the presence of other comorbidities often associated with ED (i.e., other personality disorders, complex post-traumatic disorder, substance use disorder, mood disorders, anxiety disorder, and autism spectrum disorder [1, 10]) cannot be excluded. Even though another categorical diagnosis was assessed using dimensional evaluation (post-traumatic stress disorder, PTSD), because of a growing focus in our unit on this issue, this constitutes a strong limitation to our study that will be discussed later.

Moreover, for dimensional evaluation, after the first appointment, patients undertake several self-report questionnaires to assess various psychopathological dimensions. Regarding emotion regulation, we use the DERS in its 18-items version [29]. We also use the Cognitive Emotion Regulation Questionnaire 36 items (CERQ) [30] to evaluate nine cognitive strategies for regulating emotions, both adaptive (e.g., positive refocusing, acceptance) and maladaptive (e.g., rumination, catastrophizing). In both scales, items are rated on a five-point Likert scale, with higher scores indicating greater difficulties in emotion regulation and higher use of the strategy, respectively. A DERS total score and two CERQ total scores (adaptive and non-adaptive) can be calculated by summing all the relevant dimensions.

Borderline and ADHD symptoms severity are assessed using the Borderline Symptom List – 23 items (BSL) [31] and the Conners Adult ADHD Rating Scale – 26 items (CAARS) [32]. The BSL evaluates core aspects of borderline symptomatology, each item being rated on a five-point Likert scale, with higher mean total score reflecting greater symptom severity. On the other hand, the CAARS evaluates four ADHD dimensions, including inattention, hyperactivity, impulsivity, emotional lability, and self-concept issues, each item being rated on a four-point Likert scale, with higher scores indicating greater ADHD-related difficulties. Post-traumatic stress disorder (PTSD) symptomatology and diagnosis are assessed using the Post-Traumatic Stress Disorder Checklist for DSM-5 (PCL-5), a 20-items self-report questionnaire assessing the four dimensions of PTSD (intrusions, avoidance, negative mood/cognition, and hyperarousal), with higher scores indicating higher intensity of symptoms [33].

Moreover, mentalizing abilities are assessed using the Mentalization Scale (MentS) [34], a 28-items self-report scale measuring self-mentalizing, other-mentalizing, and motivation to mentalize. Higher scores indicate greater mentalizing abilities. Finally, depressive symptomatology is assessed using the Beck Depression Inventory-II (BDI-II) [35], trait anxiety is assessed using the State-Trait Anxiety Inventory (STAI-Y) [36], and mental well-being is assessed using the World Health Organization-Five Well-Being Index (WHO-5) [37].

To note, measures of borderline, ADHD, post-traumatic stress symptoms, and mentalizing abilities were chosen because of the focus of our unit (ADHD, BPD, and slightly less PTSD), and/or because of their well-known link with emotion dysregulation.

### Statistical analysis

First, we applied descriptive statistics on the overall sample to search for aberrant values and missing data. For quantitative variables, missing data (mean = 1.2%) were handled using multiple imputation via the *mice* package in R [38]. Imputation was performed on all quantitative variables included in the analysis, and five imputed datasets were generated (m = 5). The imputed quantitative variables were reintegrated into the main dataset, allowing retention of the categorical and derived variables not included in the imputation model. Missing data for binary variables (mean = 3.15%) were imputed by the negative. Second, to identify homogeneous subgroups of patients, we performed Latent Profile Analysis (LPA) on the six subscales of the DERS. We chose the DERS scale as a basis because many previous cluster-analytic studies on ED have relied on the DERS, which allows direct comparison and integration of our results with the existing literature. Latent profile models with one to five classes were estimated using the tidyLPA package in R [39]. A model specification assuming unequal variances and varying covariances among the indicators was used to account for intercorrelations between subscales and heterogeneity in variances. Model selection was guided by several fit indices: the Akaike Information Criterion (AIC), the Bayesian Information Criterion (BIC), and the sample-size adjusted BIC (SABIC), where lower values indicate better relative fit; the Entropy index, with higher values indicating clearer classification (up to 1 for perfect separation); and the Integrated Completed Likelihood (ICL), where higher values indicate better fit with clearer class separation. Third, to compare the differences between pairs of clusters, we used Student t-test (for quantitative variables) or Chi-Squared test (for binary variables), after checking for validity conditions. Finally, given the exploratory nature of the present study, we did not adjust for multiple comparisons [40]. Statistical significance threshold was thus set at 0.05. Analyses were conducted using R software [41].

## Results

A total 349 patients were included in the analysis (239 females (68.48%), mean age = 31.37 (SD = 11.84)). In our sample, the prevalences of ADHD, BPD, and PTSD were 34.96% (inattentive 13.47%, hyperactive 1.15%, and mixed type 20.34%), 47.56%, and 50.43%, respectively. The rest of the descriptive statistics conducted on the overall sample can be found in the Supplementary Material (TableS1).

Fit indices for each model can be found in Table 1 and DERS scores and sub-scores for each model can be found in Table S2. While the three-class model yielded the lowest AIC and SABIC, the two-class solution showed the lowest BIC. Entropy values were acceptable (>0.80) across the two- and three-class models, with the four-class solution showing the highest entropy but at the cost of substantially worse BIC and ICL. Moreover, ICL consistently declined as additional classes were introduced, thereby offering no support for solutions more complex than the two-class model. In line with recommendations by Nylund and colleagues [42] and the analytic hierarchy framework proposed by Akogul & Erisoglu [43], the two-class model was retained as the best-fitting and most parsimonious solution.

**Table 1:** Fit indices for model selection

Classes	AIC	BIC	SABIC	Entropy	ICL
1	10656.43	10760.52	10674.87	1	−10760.52
2	10518.99	10731.02	10556.54	0.8444267	−10770.55
3	10492.65	10812.62	10549.31	0.8183703	−10879.32
4	10498.23	10926.15	10574.02	0.8598642	−10986.07
5	Warning*				

*AIC* Akaike Information Criterion, *BIC* Bayesian Information Criterion, *SABIC* Sample-Size Adjusted BIC, *ICL* Integrated Completed Likelihood

*In tidyLPA, a warning indicates that the solution did not converge adequately (e.g., due to very small class sizes or non-positive variance estimates), and therefore cannot be considered a reliable solution

Two clusters were thus retained based on the results of the LPA, Cluster 1 (C1, *n* = 267) and Cluster 2 (C2, *n* = 82). Descriptive statistics and between-cluster comparisons can be found in Table 2. The two clusters differed significantly across sociodemographic, clinical, and psychological variables. Compared to C2, individuals in C1 were significantly younger (C1 = 29.68, C2 = 36.87, *p* < 0.001) and more likely to be female (75.66% vs. 45.12%, *p* < 0.001). C1 also had higher rates of psychiatric hospitalization (36.7% vs. 24.39%, *p* = 0.039), past suicide attempts (46.07% vs. 15.85%, *p* < 0.001), and non-suicidal self-injury (76.03% vs. 54.88%, *p* < 0.001). Likewise, in terms of diagnosis, differences were marked: BPD was far more frequent in C1 (59.55%) than in C2 (8.54%, *p* < 0.001), as was PTSD (59.55% vs. 20.73%, *p* < 0.001), but not ADHD (e.g., for all subtypes: 32.58% vs. 42.68%, *p* = 0.093). Regarding emotion regulation, emotion dysregulation was more severe in C1 across all DERS subscales and on DERS total score (e.g., for total score, 60.15 vs. 37.55, *p* < 0.001). C1 endorsed significantly more non-adaptive strategies (49.58 vs. 37.33, *p* < 0.001), and C2 reported more adaptive ones (53.55 vs. 65.51, *p* < 0.001). Moreover, regarding mentalizing, C1 showed significantly lower self-mentalizing ability (20.58 vs. 26.87, *p* < 0.001), while no group differences were observed in other-mentalizing or motivation to mentalize. Finally, regarding the other psychopathological dimensions, C1 exhibited significantly higher borderline (1.99 vs. 0.85, *p* < 0.001), PTSD (38.28 vs. 19.29, *p* < 0.001), ADHD (e.g., for CAARS total score, 49.65 vs. 39.12, *p* < 0.001), depressive (31.75 vs. 15.08, *p* < 0.001) and trait anxiety (61.88 vs. 47.24, *p* < 0.001) symptoms, as well as lower psychological well-being (7.92 vs. 12.25, *p* < 0.001).

**Table 2:** Cluster socio-demographic characteristics and group differences

Variables	Cluster 1 (*n* = 267)	Cluster 2 (*n* = 82)	Test-value (*p*)
**Binary variables – n (%)**			
*Socio-demographics*			
Gender (female)	202 (75.66%)	37 (45.12%)	**27.10 (< 0.001)**
History of Hospitalization	98 (36.7%)	20 (24.39%)	**4.25 (0.039)**
History of NSSI	203 (76.03%)	45 (54.88%)	**13.65 (< 0.001)**
History of SA	123 (46.07%)	13 (15.85%)	**24.08 (< 0.001)**
*Diagnosis*			
BPD	159 (59.55%)	7 (8.54%)	**65.46 (< 0.001)**
ADHD	87 (32.58%)	35 (42.68%)	2.81 (0.093)
Inattentive	34 (12.73%)	13 (15.85%)	0.52 (0.469)
Hyperactive/impulsive	4 (1.5%)	0 (0%)	X
Mixed	49 (18.35%)	22 (26.83%)	2.78 (0.095)
PTSD	159 (59.55%)	17 (20.73%)	**37.82 (< 0.001)**
**Quantitative variables – mean (SD) [range]**			
*Socio-demographics*			
Age	29.68 (10.94) [16–71]	36.87 (13.00) [16–77.00]	**−4.97 (< 0.001)**
* Emotion regulation*			
DERS			
Awareness	8.70 (3.23) [3–15]	7.16 (3.19) [3–15]	**3.78 (< 0.001)**
Clarity	9.63 (3.19) [3–15]	5.45 (1.79) [3–9]	**15.05 (< 0.001)**
Goals	12.83 (2.37) [3–15]	9.71 (3.54) [3–15]	**9.19 (< 0.001)**
Impulse	10.07 (3.77) [3–15]	4.66 (1.53) [3–8]	**18.92 (< 0.001)**
Non-acceptance	9.42 (3.86) [3–15]	4.93 (1.71) [3–8]	**14.84 (< 0.001)**
Strategies	10.39 (3.19) [3–15]	5.22 (1.69) [3–9]	**19.14 (< 0.001)**
Total	60.15 (11.28) [25–90]	37.55 (9.89) [18–90]	**16.32 (< 0.001)**
CERQ			
Adaptive	53.55 (13.77) [20–94]	65.51 (13.65) [27–96]	**−6.89 (< 0.001)**
Non-adaptive	49.58 (10.60) [27–79]	37.33 (7.75) [20–56]	**9.69 (< 0.001)**
*Other variables*			
MentS			
Self	20.58 (6.17) [8–37]	26.87 (6.21) [14–40]	**−8.06 (< 0.001)**
Other	38.30 (6.64) [14–50]	38.87 (5.18) [28–50]	−0.70 (0.482)
Motivation	39.04 (6.49) [21–50]	39.33 (5.87) [24–49]	−0.35 (0.723)
BSL-23 mean score	1.99 (0.92) [0–4]	0.85 (0.57) [0–2.48.48]	**13.56 (< 0.001)**
CAARS			
Inattentive	16.05 (5.17) [1–24]	14.61 (6.07) [0–24]	**2.11 (0.036)**
Hyperactive	10.08 (4.25) [0–18]	8.84 (4.43) [0–18]	**2.30 (0.022)**
Impulsive	10.42 (3.96) [1–18]	5.75 (3.08) [0–12]	**9.82 (< 0.001)**
Self-concept	13.10 (3.57) [1–18]	9.92 (3.74) [1–16]	**6.99 (< 0.001)**
Total	49.65 (11.72) [9–75]	39.12 (12.98) [5–66]	**6.94 (< 0.001)**
PCL total score	38.28 (21.64) [0–80]	19.29 (16.69) [0–77]	**7.30 (< 0.001)**
BDI total score	31.75 (12.55) [1–61]	15.08 (9.67) [0–39]	**11.06 (< 0.001)**
STAI trait score	61.88 (9.89) [27–80]	47.24 (10.31) [20–68]	**11.61 (< 0.001)**
WHO-5 total score	7.92 (4.71) [0–20]	12.25 (4.80) [0–24]	**−7.24 (< 0.001)**

*ADHD* Attention Deficit Hyperactivity Disorder, *BDI* Beck Depression Inventory, *BPD* Borderline Personality Disorder, *BSL* Borderline Symptom List 23 items, *CAARS* Conners’ Adult ADHD Rating Scale, *CERQ* Cognitive Emotion Regulation Questionnaire – 36 items, *DERS* Difficulties in Emotion Regulation Scale – 18 items, *MentS* Mentalization Scale, *NSSI* Non-suicidal Self-injury, *PCL* Post-traumatic stress disorder checklist for DSM-5, *PTSD* Post Traumatic Stress Disorder, *SA* Suicidal Attempt, *STAI* State and Trait Anxiety Inventory, *WHO-5* The World Health Organization-Five Well-Being Index

## Discussion

In this study, we aimed to identify subgroups in a clinically heterogeneous population of adult patients presenting ED and to explore their characteristics and specificities. We applied latent profile analyses (using the DERS sub-scores as bases) and identified two clusters of patients, with marked differences across sociodemographic, emotional, and psychopathological dimensions. Cluster 1 individuals demonstrated more severe emotion dysregulation on every dimension, higher use of non-adaptive emotion regulation strategies and lower self-mentalizing abilities, more severe comorbid psychopathology, and lower well-being. On the other hand, Cluster 2 individuals demonstrate a comparatively preserved clinical and psychological profile, with better emotion regulation and self-mentalizing abilities, less comorbid psychopathology, and higher well-being. Although exploratory, we believe these findings provide valuable insights on the identification and description of subgroups of patients struggling with ED.

First, our results significantly align with previous studies conducted in adult clinical populations showing that subgroups of patients classified based on their emotion regulation difficulties tend to arrange along a severity continuum, from preserved to severely dysregulated profiles, with concomitant variations in terms of psychiatric comorbidities and psychosocial functioning [18, 24, 25]. However, contrary to most of these studies that consistently identified three classes, our analyses yielded a two-class solution. In our opinion, this discrepancy can be understood in two main ways. First, it could be linked to the way the number of clusters were determined in each study. In our research, the selection of the two-class model was based on the lowest BIC (following recommendations of Nylund and colleagues [42]) and on the analytic hierarchy framework proposed by Akogul & Erisoglu [43]. This approach was the same used in the only other clinical study that found a two-class solution [24]. On the other hand, studies founding a three-class solution either used a two-step cluster analysis (with its inherent limitations, including the absence of estimates for how the model fits the observed data) [23] or latent profile analysis using all the classic estimates but without using the analytic hierarchy framework mentioned earlier [25]. Second, this discrepancy could also be linked to the type of clinical population used. For example, the two LPA studies conducted on BPD patients found a three-class solution [18, 25], whereas the other LPA study was conducted on patients with binge-spectrum eating disorders and found a two-class solution [24]. The fact that our study was conducted on a clinically heterogeneous population of patients could thus have influenced the number of classes found. Altogether, despite this discrepancy in terms of number of classes, we believe that our results support the growing literature stating that subgroups of patients within the ED dimension tend to arrange along a severity continuum.

Second, and consistently with this perspective, our findings also indicate that these clusters do not align with specific diagnostic categories such as BPD, ADHD, or PTSD. Indeed, ADHD prevalence was statistically similar between clusters regardless of severity and, even though BPD and PTSD were found in both clusters, their prevalences were significantly increased in the more dysregulated cluster (C1). Such discrepancy between cluster analysis results and classic diagnostic category has already been described in psychiatry (e.g [44]).,, and similar results were found in a cluster-analytic study conducted in a clinically heterogeneous adolescent population [23]. Altogether, our findings further emphasize the limitations of categorical diagnostic frameworks in capturing the heterogeneity and complexity of ED.

Third, we found that as the severity of ED, associated comorbid psychopathology and impaired well-being increased from C2 to C1, self-mentalizing abilities (but not other-mentalizing or motivation to mentalize) decreased. This specific role of *self-*mentalizing can be compared to past studies showing that adaptive *and* maladaptive emotion regulation can be predicted only by self-focused mentalizing [45]. Moreover, we also found a concomitant decrease in the use of adaptive emotional regulation strategies and a concomitant increase in the use of non-adaptive emotion regulation strategies. In other words, the more emotionally dysregulated and impaired a patient is, the less he/she tends to self-mentalize, and the higher he tends to use non-adaptive strategies to regulate emotions. These results align with DBT’s and MBT’s conceptualization and treatment of ED. Indeed, DBT links ED to the tendency to rely on non-adaptive emotion regulation strategies and offers to improve it through the development of adaptive emotion regulation skills, while MBT links ED to lower abilities to mentalize Self and others and offers to improve it through mentalizing training [5, 6]. This can be compared to previous research suggesting the idea that interventions targeting ED, including DBT and MBT, could be relevant beyond the sole BPD diagnosis [1]. Indeed, by showing that the mechanisms targeted by DBT and MBT—use of maladaptive strategies and self-mentalizing difficulties—are also central in a broader, clinically heterogeneous population of patients with ED, we believe that our results therefore contribute to this line of reasoning. However, this interpretation remains exploratory, and future studies are needed to directly evaluate the effectiveness of these approaches in clinically heterogeneous samples.

Our study has several limitations that our readers should be aware of. First, the sample was drawn from a specialized outpatient unit primarily focusing on BPD and ADHD. Even though many patients were referred to for ED difficulties irrespective of a specific diagnosis, the unit’s specialization likely introduced selection bias, as suggested by the high prevalence of BPD and ADHD in our sample, or by the characteristics of patients in Cluster 2. Indeed, patients in this subgroup reported only mild ED, which seems unexpected given the clinical focus of our unit. This may be explained by the ADHD orientation of the unit, that may have led to the inclusion of patients with lower levels of ED whose main referral concern was attentional difficulties. Moreover, other diagnoses often associated with ED were not categorically assessed, and thus we did not have any information regarding their prevalence in our sample. Finally, our sample included all individuals who were referred to our unit, irrespective of the motive (e.g., diagnostic only, diagnostic and treatment) or the mode of referral (self-referred, clinician-referred). All these aspects limit the generalizability of our findings to broader clinical or community-based populations with mental disorders, and the conclusions we can draw (notably on the role of mentalizing abilities, which are known to be influenced by many psychiatric comorbidities). Second, the fact that we used only the DERS to assess emotion dysregulation, and that we only compared our results to studies also using the DERS, also limit the generalizability of our results, as ED can be evaluated through numerous other scales [21]. Moreover, another psychometric limitation rely on the sole reliance on self-report measures, assessed at one time only, which may have increased the risk of measurement bias and common method variance and led to false positive associations [46]. Integrating clinician-rated assessments and longitudinal follow-ups would strengthen future research. Third, the exploratory nature of this study and the lack of predefined hypotheses impede the possibility to draw definite conclusion on the topics addressed. However, despite these limitations, our study is the first to be conducted in a clinically heterogeneous adult population, extending prior research focused on single diagnostic categories or in adolescent populations. Its significant sample size (*N* = 349), its multidimensional approach integrating both dimensional and categorical constructs, along with the use of validated statistical techniques such as LPA, enhance this study’s relevance and utility in advancing knowledge on ED as a dimensional construct.

## Conclusion

This study provides further evidence on the existence and specificities of subgroups in patients suffering with ED. Using LPA conducted on DERS sub-scores, we identified two distinct subgroups that did not align with classic diagnostic categories, but were arranged along a severity continuum. While exploratory, our findings also suggest that core mechanisms targeted by evidence-based psychotherapeutic interventions such as DBT and MBT may be pertinent across a broader transdiagnostic spectrum. Future studies should replicate these results in more generalizable sample, with the integration of multi-method and longitudinal designs, and examine the clinical implications of tailoring interventions to ED severity profiles.

## Supplementary Information

Supplementary Material 1.

## Data Availability

All data, analysis code, and research materials have been made publicly available at the Open Science Framework (OSF) and can be accessed at: https://osf.io/7q3ab/?view_only=398ac148be344a99a98c81d5cd846bdd.

## Abbreviations

ADHD: Attention-Deficit/Hyperactivity Disorder
AIC: Akaike Information Criterion
BDI: Beck Depression Inventory
BIC: Bayesian Information Criterion
BPD: Borderline Personality Disorder
BSL: Borderline Symptom List 23 items
CAARS: Conners’ Adult ADHD Rating Scale
CERQ: Cognitive Emotion Regulation Questionnaire
DBT: Dialectical Behavior Therapy
DERS: Difficulties in Emotion Regulation Scale
ED: Emotion Dysregulation
ICL: Integrated Completed Likelihood
LPA: Latent Profile Analysis
MBT: Mentalization-Based Therapy
MentS: Mentalization Scale
PCL: PTSD Checklist for DSM-5
PTSD: Post-Traumatic Stress Disorder
SABIC: Sample-size Adjusted BIC
STAI-Y: State-Trait Anxiety Inventory - Form Y
WHO-5: World Health Organization - Five Well-Being Index
