# Supplementary material for: Subgroups of emotion dysregulation in adults: a latent profile analysis in a clinically heterogeneous population
Source: Borderline Personal Disord Emot Dysregul. 2025 Dec 4;12:50. doi: 10.1186/s40479-025-00319-x (PMC12679781; doi:10.1186/s40479-025-00319-x)
Supplement: Supplementary file 1 — Supplementary Material 1. [file 40479_2025_319_MOESM1_ESM.docx]

**Supplementary Material - Subgroups of emotion dysregulation in adults: A latent profile analysis in a clinically heterogeneous population**

Martin Blay*, Amaury Durpoix, Mario Speranza, Roland Hasler, Rosetta Nicastro, Eleonore Pham, Eva Rufenacht, Luisa Weiner, Sébastien Weibel, Martin Debbané, Nader Perroud

** Corresponding Author*: Martin BLAY, MD, MSc (ADDIPSY Addictology and Psychiatry Outpatient Center, Santé Basque Développement group, 164 Avenue Jean Jaurès, Lyon, 69007, France), [martin.blay5@gmail.com](mailto:martin.blay5@gmail.com)

| **Tableau S1.** Descriptive statistics on the overall sample (n=349) | |
| --- | --- |
| **Quantitative variables** | **Mean (SD)** |
| **Age** | 31.37 (11.84) |
| **BSL mean score** | 1.73 (0.98) |
| **CAARS** |  |
| Inattentive | 15.71 (5.42) |
| Hyperactive | 9.79 (4.32) |
| Impulsive | 9.32 (4.26) |
| Self-concept | 12.35 (3.85) |
| Total | 47.18 (12.81) |
| **BDI total score** | 27.84 (13.87) |
| **STAI trait total score** | 58.44 (11.75) |
| **CERQ** |  |
| Blaming Self | 13.60 (4.11) |
| Acceptance | 13.25 (3.83) |
| Rumination | 14.18 (4.14) |
| Positive Refocusing | 7.88 (3.69) |
| Refocus on planning | 12.50 (4.35) |
| Positive Reappraisal | 11.12 (4.57) |
| Putting into perspective | 11.60 (4.03) |
| Catastrophizing | 9.72 (4.05) |
| Blaming Others | 9.20 (3.92) |
| Total adaptive | 56.36 (14.63) |
| Total nonadaptive | 46.70 (11.26) |
| **DERS** |  |
| Awareness | 8.34 (3.28) |
| Clarity | 8.65 (3.42) |
| Goals | 12.10 (2.99) |
| Impulse | 8.80 (4.09) |
| Non-acceptance | 8.36 (3.96) |
| Strategies | 9.18 (3.64) |
| Total | 54.84 (14.56) |
| **PCL total score** | 33.82 (22.08) |
| **MentS** |  |
| Self | 22.05 (6.72) |
| Others | 38.44 (6.33) |
| Motivation | 39.11 (6.34) |
| **WHO index total score** | 8.94 (5.07) |
| **Binary variables** | **n (%)** |
| **Gender (female)** | 239 (68.48%) |
| **History of Hospitalization** | 118 (33.81%) |
| **History of SA** | 136 (38.97%) |
| **History of NSSI** | 248 (71.06%) |
| **PTSD** | 176 (50.43%) |
| **ADHD** | 122 (34.96%) |
| Inattentive | 47 (13.47%) |
| Hyperactive | 4 (1.15%) |
| Mixed | 71 (20.34%) |
| **BPD** | 166 (47.56) |
| *Abbreviations : ADHD = Attention Deficit Hyperactive Disorder; BDI = Beck Depression Inventory ; BPD = Borderline Personality Disorder ; BSL = Borderline Symptom List 23 items; CAARS = Conners’ Adult ADHD Rating Scale ; CERQ = Cognitive Emotion Regulation Questionnaire – 36 items; DERS = Difficulties in Emotion Regulation Scale – 18 items ; MentS = Mentalization Scale ; NSSI = Non-Suicidal Self Injury ; PCL = Post-traumatic stress disorder checklist for DSM-5 (PCL-5) ; PTSD = Post Traumatic Stress Disorder ; SA = Suicidal Attempts ; STAI = State and Trait Anxiety Inventory ; WHO-5 = The World Health Organization-Five Well-Being Index* | |

| **Tableau S2.** DERS total and subscores in each class solution | | | | | |
| --- | --- | --- | --- | --- | --- |
| **Two-class solution** | | | | | |
| **Variables** | | **Class 1 (n=267)** | **Class 2 (n=82)** |  |  |
| Awareness | | 8.70 (3.23) | 7.16 (3.19) |  |  |
| Clarity | | 9.63 (3.19) | 5.45 (1.79) |  |  |
| Goals | | 12.83 (2.37) | 9.71 (3.54) |  |  |
| Impulse | | 10.07 (3.77) | 4.66 (1.53) |  |  |
| Non-acceptance | | 9.42 (3.86) | 4.93 (1.71) |  |  |
| Strategies | | 10.39 (3.19) | 5.22 (1.69) |  |  |
| Total | | 60.15 (11.28) | 37.55 (9.89) |  |  |
| **Three-class solution** | | | | | |
| **Variables** | **Class 1 (n=112)** | | **Class 2 (n=156)** | **Class 3 (n=81)** |  |
| Awareness | 9.46 (3.20) | | 8.03 (3.19) | 7.38 (3.18) |  |
| Clarity | 9.51 (2.97) | | 9.65 (3.42) | 5.52 (1.75) |  |
| Goals | 12.70 (2.45) | | 12.88 (2.38) | 9.76 (3.53) |  |
| Impulse | 9.78 (3.99) | | 10.13 (3.76) | 4.85 (1.64) |  |
| Non-acceptance | 5.45 (2.00) | | 12.15 (2.15) | 5.09 (1.73) |  |
| Strategies | 9.64 (3.00) | | 10.86 (3.29) | 5.30 (1.77) |  |
| Total | 55.79 (11.13) | | 62.76 (11.22) | 38.27 (10.26) |  |
| **Four-class solution** | | | | | |
| **Variables** | | **Class 1 (n=80)** | **Class 2 (n=178)** | **Class 3 (n=53)** | **Class 4 (n=38)** |
| Awareness | | 10.60 (2.94) | 8.30 (3.14) | 7.11 (2.85) | 5.47 (1.67) |
| Clarity | | 9.46 (3.34) | 9.67 (3.29) | 6.98 (1.75) | 4.47 (1.29) |
| Goals | | 11.43 (3.49) | 13.02 (2.26) | 12.84 (1.56) | 8.16 (2.91) |
| Impulse | | 9.33 (4.24) | 10.37 (3.73) | 6.00 (1.87) | 4.21 (1.32) |
| Non-acceptance | | 4.53 (1.48) | 11.65 (2.44) | 6.15 (1.59) | 4.13 (1.38) |
| Strategies | | 8.53 (3.50) | 10.93 (3.13) | 7.82 (2.26) | 4.24 (1.08) |
| Total | | 53.30 (12.41) | 62.99 (10.68) | 47.17 (7.37) | 30.58 (5.51) |
